# Subtotal versus total gastrectomy for mid- and distal gastric poorly cohesive carcinoma: a retrospective controlled study

**DOI:** 10.3389/fonc.2025.1717678

**Published:** 2025-11-24

**Authors:** Burak Dinçer, Mehmet Furkan Sağdıç, Mehmet Olgun, Ahmet Karayiğit, Gülay Bilir Dilek, Serdar Sarıdemir, Cihangir Özaslan

**Affiliations:** 1 Department of Surgical Oncology, University of Health Sciences, Gülhane School of Medicine, Ankara Oncology Training and Research Hospital, Ankara, Türkiye; 2 Department of Surgical Oncology, Ankara Etlik City Hospital, Ankara, Türkiye; 3 Department of Surgery, University of Health Sciences, Gülhane School of Medicine, Ankara Oncology Training and Research Hospital, Ankara, Türkiye; 4 Department of Pathology, University of Health Sciences, Gülhane School of Medicine, Ankara Oncology Training and Research Hospital, Ankara, Türkiye

**Keywords:** gastric cancer, Lauren’s diffuse gastric cancer, poorly cohesive carcinoma, subtotal gastrectomy, total gastrectomy

## Abstract

**Background:**

Subtotal gastrectomy is frequently performed for distally located gastric tumors and carries a lower risk of postoperative complications compared to total gastrectomy. However, due to the submucosal spread pattern and worse prognosis of poorly cohesive carcinoma (PCC), some authors advocate for routine total gastrectomy. This study aimed to compare the outcomes of subtotal versus total gastrectomy in patients with mid- and distal-located gastric PCC.

**Methods:**

This single-center retrospective study included patients who underwent resection for gastric PCC between 2012 and 2024. Exclusion criteria were systemic metastasis, palliative surgery, and tumors located in the proximal one-third of the stomach. Patients were analyzed based on demographic, clinical, pathological, and survival data.

**Results:**

A total of 154 patients were included. The median age was 62 years (range: 36–87), and 83 patients (53.9%) were male. Subtotal gastrectomy was performed in 70 patients (45.5%). The median pathological tumor diameter was 60 mm (IQR: 40–90). Over a median follow-up of 79 months, 33 locoregional and 81 systemic recurrences were observed among 146 patients, and 53 patients (36.3%) were alive at the time of last follow-up. Pathological stage was the only independent factor associated with overall survival, while the type of surgery (subtotal vs. total gastrectomy) did not significantly affect survival outcomes.

**Conclusion:**

Our study demonstrated that subtotal gastrectomy yielded oncologic outcomes similar to those achieved with total gastrectomy in mid- and distal gastric poorly cohesive carcinoma cases.

## Introduction

In epidemiological studies, a declining trend in the incidence of gastric cancer has been observed over the years (1). However, this trend differs in populations under the age of 50, in whom an increasing incidence has been reported (2). The overall decrease in incidence may be attributed to a reduced prevalence of Laurén’s intestinal-type gastric cancers, which are more common in high-incidence regions and have declined due to improvements in Helicobacter pylori (HP) eradication and changing dietary habits (3). Gastric poorly cohesive carcinoma (PCC), on the other hand, has a lower overall incidence compared to the intestinal type but tends to present at a younger age, follows a more aggressive clinical course, and exhibits a higher propensity for peritoneal metastasis (4, 5). The increased prevalence of gastric PCC (Laurén’s diffuse-type gastric cancer) in younger populations and in regions with lower overall gastric cancer incidence may explain the rising incidence observed in patients under 50 years of age. Although terms such as signet ring cell carcinoma (SRC) or PCC are used for diffuse-type gastric cancers, there is ongoing debate among pathological classification systems. Consequently, consensus efforts have been made to standardize terminology in the literature (6).

Currently, surgical resection remains the only potentially curative treatment option for gastric cancer. In cases of locally advanced disease, subtotal or total gastrectomy combined with D2 lymph node dissection is typically performed (7). Due to the diffuse infiltration pattern of gastric PCC, some guidelines recommend wider macroscopic resection margins, and intraoperative frozen section analysis is advised when necessary. Because of its aggressive nature and higher risk of microscopic margin positivity, total gastrectomy is more frequently performed in this patient group (8, 9). However, total gastrectomy is associated with higher morbidity and mortality rates compared to subtotal gastrectomy, and long-term oncological outcomes appear similar between the two approaches (10, 11). Given that gastric PCC is underrepresented in many of these studies and data in the literature remain limited, the optimal surgical approach for this tumor subtype remains a matter of debate.

In this study, we aimed to compare the outcomes of total versus subtotal gastrectomy in patients undergoing curative surgery for gastric PCC and to evaluate the oncological adequacy of subtotal gastrectomy in this patient population.

## Materials and methods

### Study population and selection criteria

In this study, archival records of 878 patients diagnosed with gastric cancer at our center were retrospectively reviewed. A total of 154 patients who underwent subtotal or total gastrectomy for histologically confirmed gastric PCC between 2012 and 2024 were included in the analysis. The study was conducted in accordance with the Declaration of Helsinki and approved by the Non-Interventional Clinical Research Ethics Committee of Ankara Oncology Training and Research Hospital (02.01.2025 – No: 2024-12/198).

Patients aged over 18 years who underwent curative surgery for mid- or distal-located gastric cancer and were pathologically diagnosed with PCC were included. Patients with non-PCC gastric cancer, tumors located in the proximal third of the stomach, those who underwent palliative surgery, had distant metastases or an active malignancy other than gastric cancer, or had incomplete follow-up data or missing data were excluded. Due to the limited number of patients who underwent laparoscopic surgery, most of whom had subtotal gastrectomy, laparoscopic cases were not included in the study. Patients were evaluated based on age, sex, family history, American Society of Anesthesiologists (ASA) physical status score (12), tumor localization, neoadjuvant and adjuvant treatments, surgical details, complications, pathological tumor features, recurrence rates, and survival outcomes.

For pathological classification, the 8th edition of the American Joint Committee on Cancer (AJCC) tumor-node-metastasis staging system for gastric cancer, the 2019 World Health Organization (WHO) Classification of Tumours of the Digestive System (5th edition), and the 2019 consensus report by the European Chapter of the International Gastric Cancer Association were used (6, 13, 14).

In survival analyses, the date of surgery was considered the starting point. Locoregional disease-free survival (LDFS) was defined as the time from surgery to locoregional recurrence, systemic disease-free survival (SDFS) as the time to systemic recurrence, and overall survival (OS) as the time to death. In cases without events, the survival time was calculated as the period from surgery to the last follow-up.

Postoperative complications were assessed using the Clavien-Dindo classification (15). Complications classified as Clavien-Dindo grade 3a or higher were considered major complications, while the rest were defined as minor.

### Outcome measures

In our study, overall survival (OS) and the factors influencing OS were determined as the primary outcome. Locoregional disease-free survival (LDFS), systemic disease-free survival (SDFS), and the factors affecting these outcomes were determined as the secondary outcomes.

### Surgical techniques

At our center, the standard surgical approach for gastric cancer includes either total or distal gastrectomy, determined based on tumor location. For curative lymphadenectomy, a D2 lymph node dissection was performed as described in the Japanese Gastric Cancer Treatment Guidelines 2021 (6th edition) (16). While laparoscopic surgery was used for eligible patients, open surgery was preferred in cases deemed unsuitable for laparoscopy due to factors such as prior abdominal surgeries, locally advanced tumors, or comorbidities.

### Adjuvant treatments and follow-up

Gastric cancer cases were discussed in a multidisciplinary tumor board pre- and postoperatively, and decisions regarding neoadjuvant and/or adjuvant chemotherapy (CT) and/or radiotherapy (RT) were made accordingly. Postoperatively, patients were followed every three months for the first five years with physical examinations and tumor markers, and every six months with radiologic imaging. In clinically stable patients, the follow-up intervals were extended. In cases of local recurrence, systemic staging was performed, and re-resection was planned in selected cases with isolated local recurrence. Patients with systemic recurrence received systemic chemotherapy.

### Outcome measures

The primary outcome was to evaluate the impact of subtotal versus total gastrectomy on LDFS, SDFS, and OS in patients who underwent curative surgery for mid- or distal-located gastric PCC. Secondary outcomes included complication rates and rates of local or systemic recurrence during follow-up based on surgical approach.

### Statistical analysis

Statistical analysis was performed using SPSS^®^ (Statistical Package for the Social Sciences) version 25.0 (IBM Corp., Armonk, NY, USA) and Jamovi (The jamovi project (2025). *jamovi* (Version 2.6) [Computer Software]. Retrieved from https://www.jamovi.org). Descriptive statistics, including count, percentage, and median, were used to summarize the study data. The normality of continuous variables was assessed using the Shapiro-Wilk test. Normally distributed continuous variables were presented as mean ± standard deviation, while non-normally distributed variables were expressed as median and interquartile range (IQR). Survival data were analyzed using Kaplan-Meier curves, and factors affecting survival were assessed with the log-rank test. Categorical variables were analyzed using Pearson Chi-square test or Fisher’s Exact test, while continuous variables were compared using Student’s t-test or the Mann-Whitney U test. Parameters found to be significant or nearly significant (p<0.1) in the univariate analysis were included in a multivariate Cox proportional hazards regression model. The proportional hazards assumption was assessed using Schoenfeld residuals for each covariate. All p-values were two-sided, and results were considered statistically significant at p<0.05 with a 95% confidence interval.

## Results

A total of 154 patients who underwent curative resection with a diagnosis of PCC were included in our study. Subtotal gastrectomy was performed in 70 patients (45.5%), and total gastrectomy was performed in 84 patients (54.5%), with all patients undergoing D2 lymphadenectomy. The median age of the patients was 62 years (range: 36–87), and 83 patients (53.9%) were male. A family history of cancer was present in 29.9% of the patients. The ASA score was 2 in 91 patients. Tumors were located in the gastric corpus in 101 patients. Neoadjuvant CT was administered to 20 patients, while no patients received neoadjuvant RT. In the postoperative period, 75.3% of patients received adjuvant CT and 37% received adjuvant RT. Tumors located in the antrum were significantly more common in patients who underwent subtotal gastrectomy (p<0.001). No significant differences were observed between the groups in other demographic and clinical parameters (**Table 1**).

**Table 1:** Demographic and clinical characteristics.

Variables	All patients (N = 154)	Subtotal gastrectomy (N = 70)	Total gastrectomy (N = 84)	P-value
Age (Years, Median, Range)	62 (36-87)	60 (36-87)	63 (36-84)	*0.519^a^*
Sex (N, %)				
Female	71 (46.1%)	28 (40%)	43 (51.2%)	*0.165^b^*
Male	83 (53.9%)	42 (60%)	41 (48.8%)	
Family history of cancer (N, %)				
No	108 (70.1%)	48 (68.6%)	60 (71.4%)	*0.700^b^*
Yes	46 (29.9%)	22 (31.4%)	24 (28.6%)	
ASA score (N, %)				
ASA 1	3 (1.9%)	1 (1.4%)	2 (2.4%)	*0.792^c^*
ASA 2	91 (59.1%)	44 (62.9%)	47 (56%)	
ASA 3	59 (38.3%)	25 (35.7%)	34 (40.5%)	
ASA 4	1 (0.6%)	0 (0%)	1 (1.2%)	
Tumor localization (N, %)				
Corpus	101 (65.6%)	20 (28.6)	81 (96.4%)	***<0.001^c^***
Antrum	53 (34.4%)	50 (71.4%)	3 (3.6%)	
Neoadjuvant CT (N, %)				
No	134 (87%)	62 (88.6%)	72 (85.7%)	*0.599^b^*
Yes	20 (13%)	8 (11.4%)	12 (14.3%)	
Adjuvant CT (N, %)				
No	38 (24.7%)	22 (31.4%)	16 (19%)	*0.076^b^*
Yes	116 (75.3%)	48 (68.6%)	68 (81%)	
Adjuvant RT (N, %)				
No	97 (63%)	40 (57.1%)	57 (67.9%)	*0.170^b^*
Yes	57 (37%)	30 (42.9%)	27 (32.1%)	

^a^Mann-Whitney U-test, ^b^Chi-square test, ^c^Fisher’s exact test.

ASA, American society of anesthesiologists; CT, Chemotherapy; RT, Radiotherapy.

All p-values less than 0.05 were bold.

The median length of hospital stay was 11 days (IQR 9–14). Surgical complications occurred in 37 patients (24%), and major complications (Clavien-Dindo grade 3a or higher) were observed in 22 patients (14.3%). Postoperative 30-day mortality due to surgery-related complications occurred in 8 patients (5.2%). Although the 30-day postoperative mortality was higher in the total gastrectomy group, the difference was not statistically significant (p=0.497) (**Table 2**).

**Table 2:** Surgical and pathological parameters.

Variables	All patients (N = 154)	Subtotal gastrectomy (N = 70)	Total gastrectomy (N = 84)	P-value
Length of Stay (Days, Median, IQR)	11 (9-14)	11 (10-14)	12 (9-13)	*0.937^a^*
Complications (N, %)				
No complications	117 (76%)	56 (80%)	61 (72.6%)	*0.380^b^*
Minor complications	15 (9.7%)	7 (10%)	8 (9.5%)	
Major Complications	22 (14.3%)	7 (10%)	15 (17.9%)	
Complication details (N, %)				
No complications	117 (76%)	56 (80%)	61 (72.6%)	*0.285^c^*
Grade 1	10 (6.5%)	4 (5.7%)	6 (7.1%)	
Grade 2	5 (3.2%)	3 (4.3%)	2 (2.4%)	
Grade 3a	6 (3.9%)	1 (1.4%)	5 (6%)	
Grade 3b	4 (2.6%)	3 (4.3%)	1 (1.2%)	
Grade 4a	4 (2.6%)	0 (0%)	4 (4.8%)	
Grade 4b	0 (0%)	0 (0%)	0 (0%)	
Grade 5 (Postoperative mortality)	8 (5.2%)	3 (4.3%)	5 (6%)	
First 30-day postoperative mortality (N, %)				
No	146 (94.8%)	67 (95.7%)	79 (94%)	*0.729^c^*
Yes	8 (5.2%)	3 (4.3%)	5 (6%)	
Tumor Diameter (mm, Median, IQR)	60 (40-90)	50 (30-60)	75 (48-103)	***<0.001^a^***
Pathological diagnosis (N, %)				
PCC-NOS	96 (62.3%)	42 (60%)	54 (64.3%)	*0.614^b^*
PCC with SRC component	26 (16.9%)	11 (15.7%)	15 (17.9%)	
SRCC	32 (20.8%)	17 (24.3%)	15 (17.9%)	
Degree of differentiation (N, %)				
Well	16 (10.4%)	13 (18.6%)	3 (3.6%)	***0.007^c^***
Moderate	46 (29.9%)	21 (30%)	25 (29.8%)	
Poor	92 (59.7%)	36 (51.4%)	56 (66.7%)	
Pathological stage (N, %)				
Stage 0	2 (1.3%)	0 (0%)	2 (2.4%)	***<0.001^c^***
Stage 1A	19 (12.3%)	14 (20%)	5 (6%)	
Stage 1B	2 (1.3%)	1 (1.4%)	1 (1.2%)	
Stage 2A	6 (3.9%)	4 (5.7%)	2 (2.4%)	
Stage 2B	15 (9.7%)	10 (14.3%)	5 (6%)	
Stage 3A	37 (24%)	17 (24.3%)	20 (23.8%)	
Stage 3B	31 (20.1%)	16 (22.9%)	15 (17.9%)	
Stage 3C	42 (27.3%)	8 (11.4%)	34 (40.5%)	
Surgical margins (N, %)				
Negative	141 (91.6%)	61 (87.1%)	80 (95.2%)	*0.086^c^*
Positive	13 (8.4%)	9 (12.9%)	4 (4.8%)	
LVI (N, %)				
Absent	69 (44.8%)	41 (58.6%)	28 (33.3%)	***0.002^b^***
Present	85 (55.2%)	29 (41.4%)	56 (66.7%)	
PNI (N, %)				
Absent	69 (44.8%)	43 (61.4%)	26 (31%)	***<0.001^a^***
Present	85 (55.2%)	27 (38.6%)	58 (69%)	

^a^Mann-Whitney U-test, ^b^Chi-square test, ^c^Fisher’s exact test.

IQR, Interquartile range; LVI, Lymphovascular invasion; NOS, Not otherwise specified; PCC, Poorly cohesive carcinoma; PNI, Perineural invasion; SRC, Signet-ring cell; SRCC, Signet-ring cell carcinoma.

Clavien-Dindo classification was utilized for complication grading. Clavien-Dindo Grade 3A and higher considered as major complications.

All p-values less than 0.05 were bold.

The median pathological tumor size was 60 mm (IQR 40–90). The most common histological subtype was PCC-not otherwise specified (NOS), accounting for 62.3% of cases. Poor differentiation was observed in 92 patients (59.7%). The most common pathological stage was stage 3C, observed in 42 patients (27.3%). Surgical margin positivity was present in 13 patients (8.4%). No secondary surgery was performed in this R1 resection group; instead, appropriate adjuvant therapies were administered. Tumors in the total gastrectomy group were significantly larger, more poorly differentiated, at a more advanced stage, and showed a higher rate of lymphovascular and perineural invasion (p<0.001, p=0.007, p<0.001, p=0.002, and p<0.001, respectively) (**Table 2**).

**Figure 1:**
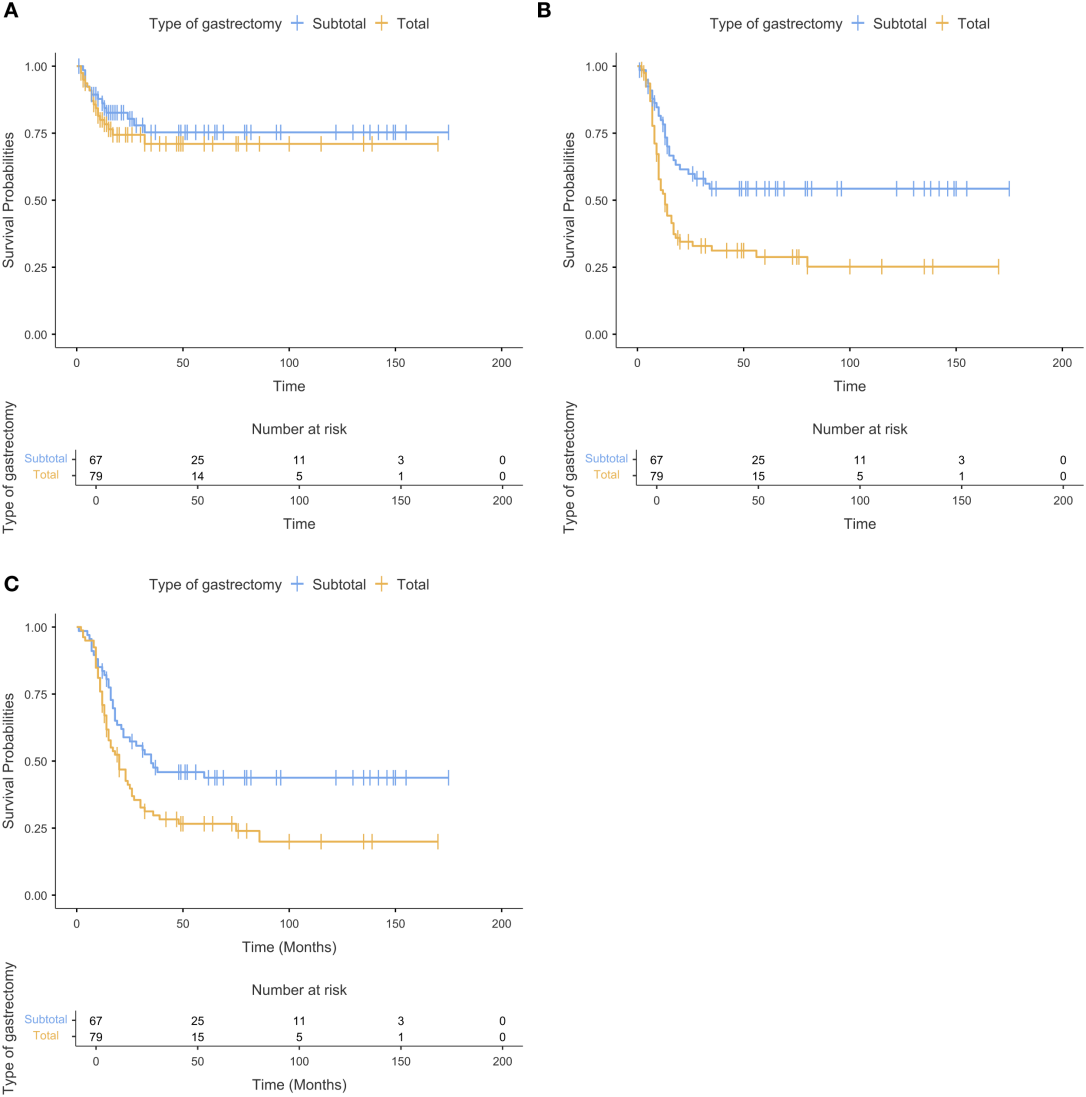
Kaplan-Meier curves demonstrating **(a)** locoregional disease-free survival, **(b)** systemic disease-free survival, and **(c)** overall survival in patients who underwent subtotal or total gastrectomy for gastric poorly cohesive carcinoma.

During a median follow-up of 79 months, 33 locoregional and 81 systemic recurrences were observed in 146 patients. At the time of last follow-up, 53 patients (36.3%) were alive (8 patients who died within 30 days postoperatively due to surgical complications were excluded from the survival analysis). Systemic recurrence and mortality rates were significantly higher in the total gastrectomy group (p=0.002 and p=0.021, respectively), whereas locoregional recurrence rates were similar between the groups (p=0.650). LDFS was comparable between the subtotal and total gastrectomy groups (p=0.440). However, both SDFS and OS were significantly lower in the total gastrectomy group (p=0.001 and p=0.009, respectively) (**Table 3**, **Figure 1**).

**Table 3:** Follow-up and survival times (Postoperative mortalities excluded).

Variables	All patients (N = 146)	Subtotal gastrectomy (N = 67)	Total gastrectomy (N = 79)	P-value
Locoregional recurrence (N, %)				
No	113 (77.4%)	53 (79.1%)	60 (75.9%)	*0.650^a^*
Yes	33 (22.6%)	14 (20.9%)	19 (24.1%)	
Locoregional DFS (Months, Median, IQR)	NR (NR-NR)	NR (NR-NR)	NR (NR-NR)	*0.440^b^*
Systemic recurrence (N, %)				
No	65 (44.5%)	39 (58.2%)	26 (32.9%)	***0.002^a^***
Yes	81 (55.5%)	28 (41.8%)	53 (67.1%)	
Systemic DFS (Months, Median, IQR)	17 (14-80)	NR (24-NR)	13 (10-18)	***0.001^b^***
Vital status (N, %)				
Alive	53 (36.3%)	31 (46.3%)	22 (27.8%)	***0.021^a^***
Deceased	93 (63.7%)	36 (53.7%)	57 (72.2%)	
Overall Survival (Months, Median, IQR)	24 (19-35)	35 (22-NR)	20 (15-26)	***0.009^b^***

^a^Chi-square test, ^b^Log-rank test.

DFS, Disease-free survival; IQR, Interquartile range; NR, Not reached.

All p-values less than 0.05 were bold.

Variables found to be statistically significant or borderline significant (p<0.1) in univariate analysis were further evaluated using multivariate Cox regression analysis. Only pathological stage was identified as an independent factor affecting OS, while surgical approach (subtotal vs. total gastrectomy) was not found to be an independent predictor of survival (**Table 4**).

**Table 4:** Factors associated with LDFS, SDFS and OS (multivariate analysis).

Variables	LDFS			SDFS			OS		
Type of surgery	HR	%95-CI	*P-value*	HR	%95-CI	*P-value*	HR	%95-CI	*P-value*
Subtotal Gastrectomy	1 (Ref)			1 (Ref)			1 (Ref)		
Total Gastrectomy	1.22	0.38-3.97	*0.740*	1.05	0.51-2.18	*0.888*	1.04	0.53-2.06	*0.904*
Tumor localization	HR	%95-CI	*P-value*	HR	%95-CI	*P-value*	HR	%95-CI	*P-value*
Antrum	1 (Ref)			1 (Ref)			1 (Ref)		
Corpus	0.74	0.23-2.37	*0.607*	1.27	0.60-2.68	*0.530*	1.03	0.52-2.03	*0.937*
Degree of differentiation	HR	%95-CI	*P-value*	HR	%95-CI	*P-value*	HR	%95-CI	*P-value*
Well	1 (Ref)			1 (Ref)			1 (Ref)		
Moderate	2.55	0.27-23.88	*0.412*	Inf	0-Inf	*0.996*	2.59	0.56-11.96	*0.222*
Poor	2.50	0.28-22.48	*0.414*	Inf	0-Inf	*0.996*	2.68	0.60-11.94	*0.197*
LVI	HR	%95-CI	*P-value*	HR	%95-CI	*P-value*	HR	%95-CI	*P-value*
No	1 (Ref)			1 (Ref)			1 (Ref)		
Yes	0.70	0.30-1.66	*0.417*	1.13	0.63-2.04	*0.680*	0.89	0.51-1.57	*0.697*
PNI	HR	%95-CI	*P-value*	HR	%95-CI	*P-value*	HR	%95-CI	*P-value*
No	1 (Ref)			1 (Ref)			1 (Ref)		
Yes	1.03	0.42-2.57	*0.941*	1.14	0.63-2.08	*0.668*	1.36	0.78-2.38	*0.277*
Pathological Stage	HR	%95-CI	*P-value*	HR	%95-CI	*P-value*	HR	%95-CI	*P-value*
Stage 0/1A/1B	1 (Ref)			1 (Ref)			1 (Ref)		
Stage 2A/2B	0.47	0.04-5.56	*0.548*	Inf	0-Inf	*0.996*	4.03	0.87-19.89	*0.087*
Stage 3A	2.93	0.55-15.69	*0.210*	Inf	0-Inf	*0.996*	6.71	1.48-30.49	***0.014***
Stage 3B	6.29	1.10-35.99	***0.039***	Inf	0-Inf	*0.996*	15.42	3.23-73.67	***0.001***
Stage 3C	5.08	0.92-28.01	*0.062*	Inf	0-Inf	*0.995*	17.47	3.80-80.36	***<0.001***

Multivariate Cox regression analysis.

CI, Confidence interval; HR, Hazard ratio; Inf, Infinite; LDFS, Locoregional disease-free survival; LVI, Lymphovascular invasion; OS, Overall survival; PNI, Perineural invasion; Ref, reference; SDFS, Systemic disease-free survival.

All p-values less than 0.05 were bold.

## Discussion

Surgery plays a central role in the current treatment approach to gastric cancer, with R0 resection offering curative potential in locoregional disease. Given their more aggressive clinical course compared to classical intestinal-type gastric cancers, PCC cases are often considered more suitable for total gastrectomy according to several publications and guidelines (8, 9). However, studies comparing the outcomes of subtotal and total gastrectomy in this specific patient population remain limited. In a multicenter study by M. Boubaddi et al. (17), it was reported that subtotal gastrectomy for distally located PCC cases yields comparable oncologic outcomes with fewer complications. In our study, not only antrum-located tumors but also mid-gastric PCCs that were technically amenable to subtotal gastrectomy were analyzed. Multivariate analysis showed that the type of gastrectomy (subtotal vs. total) had no significant impact on survival.

It has been well documented that complications following gastric cancer resection are associated with poorer prognosis, as they increase the risk of recurrence and may delay adjuvant treatments (18–21). In our study, although major complications were observed more frequently in patients who underwent total gastrectomy, the difference was not statistically significant. Based on both current literature and our findings, subtotal gastrectomy may be associated with fewer complications and may contribute positively to survival in appropriately selected patients.

Tumor stage remains one of the most critical factors guiding treatment in gastric cancer (22, 23). In our study, total gastrectomy were more commonly performed in patients with more advanced stage, poorly differentiated tumors, and those with lymphovascular and perineural invasion. This may reflect a preference for total gastrectomy in order to better adhere to oncologic principles and achieve wider resection margins, potentially improving local control. However, our multivariate analysis demonstrated that the type of gastrectomy is not adversely affect prognosis. The only independent prognostic factor for overall survival was pathological stage, consistent with findings in the literature.

The primary limitations of our study include its retrospective design and single-center setting. Additionally, patients undergoing total gastrectomy tended to have more advanced, poorly differentiated, and biologically aggressive tumors, which could bias comparisons between subtotal and total gastrectomy outcomes. The relatively small number of patients receiving neoadjuvant therapy also limits the generalizability of our findings. Also, the inclusion of only patients who underwent open surgery in our study limits the evaluation of the potential impact of laparoscopy on prognosis in these cases. In addition, due to a lack of data, the impact of surgical margin distance on survival could not be evaluated, and further studies are needed to determine the optimal surgical margin in these patients.

## Conclusions

Based on our findings, subtotal gastrectomy may be a feasible option in mid and distal gastric poorly cohesive carcinoma cases, offering comparable oncologic outcomes. Prospective studies comparing subtotal and total gastrectomy and minimal invasive techniques in this patient population are warranted to further enrich the current literature.

## Data Availability

The datasets generated and analyzed during the current study are not publicly available but are available from the corresponding author on reasonable request. Requests to access these datasets should be directed to burak-dincer@hotmail.com.
